# Proteomic landscapes of post-COVID condition: biomarkers and translational pathways

**DOI:** 10.3389/fimmu.2026.1783192

**Published:** 2026-06-24

**Authors:** Amit Bansal

**Affiliations:** Department of Clinical Science, University of Bergen, Bergen, Norway

**Keywords:** biomarkers, chronic infectious disease, long COVID, post-COVID condition, precision medicine, proteomics

## Abstract

Post−COVID condition is a heterogeneous, multi−system sequela of SARS−CoV−2 infection that imposes substantial socioeconomic burden and currently needs validated diagnostic biomarkers or established therapeutic pathways. This brief communication synthesises blood-based proteomic and targeted biomarker evidence and organises it into three overlapping pathophysiological domains: persistent immune dysregulation (IL-6, IL-20, MCP−1 and TNF- α), endothelial dysfunction and disordered haemostasis (VEGF-A, P-selectin, vWF, ICAM−1 and D−dimer); and neurological injury (NFL, GFAP, NAAA, LXN, NBL1, HAGH). Across studies, no single protein provides adequate diagnostic performance; phenotype−linked multi−analyte panels show the greatest promise. Researching post-COVID conditions requires harmonised case definitions, cross-platform validation, and the integration of coagulation assays and extracellular vesicle profiling to improve tissue signal detection.

## Introduction

The global burden of post-COVID condition (PCC) encompasses persistent fatigue, dyspnoea, cognitive and autonomic dysfunction, and reduced quality of life (1–4). PCC imposes macroeconomic and individual-level burdens, with heterogeneous symptom clusters and reduced quality of life (1, 3). Despite the proliferation of studies, clinical translation has been impeded by heterogeneous case definitions, variable sampling windows, assay nonuniformity, and small cohorts (1–4). Proteomics offers a system-level lens on disease biology, enabling discovery of coordinated pathway disturbances and practical biomarker panels. Here, I adopt the World Health Organization Delphi case definition (symptoms usually 3 months from onset, lasting ≥2 months, not explained by alternative diagnoses) (5), as a common framework for PCC, and synthesise reproducible protein patterns mapping to three interwoven domains: persistent immune activation, endothelial/vascular dysfunction with coagulopathy, and neurological injury. I emphasise convergent signals across unbiased proteomic screens and targeted assays, link them to dominant clinical phenotypes, and articulate a roadmap for clinical implementation, including standardized sampling, cross-platform verification, and prospective validation of multi-analyte panels. Identifying reproducible blood protein biomarkers is important for improving patient stratification, mechanistic insight, and therapeutic monitoring. This manuscript aims to organise PCC-related dysregulated signals into immune/inflammatory, endothelial/coagulation, and neurological injury domains, highlighting emerging evidence on reproducible proteins and their clinical correlates.

## Methods

This Brief Communication was undertaken as a focused narrative review designed to synthesise current knowledge on proteomic biomarkers associated with PCC. The Scale for the Assessment of Narrative Review Articles (SANRA) was used as a reporting guide to improve clarity and structure (6); however, no formal risk−of−bias assessment or quantitative synthesis was performed, consistent with the scope of a rapid narrative review. The primary search strategy involved structured database searches, principally MEDLINE (Ovid) and Embase (Ovid), to identify articles in the English-language up to 23 May 2026. Search terms were applied in various combinations and are described in **Table 1**. Additional articles were identified through citation tracking of retrieved papers and consultation of authoritative reports issued by international organisations and research institutions. Studies were eligible for inclusion if they adhered to the recently established clinical definition of PCC, with particular emphasis placed on translational relevance. Studies were prioritised based on relevance to blood−based proteomics, adherence to established PCC definitions, cohort size, and availability of independent validation, where reported. Conflicting findings across studies were described narratively rather than reconciled quantitatively. Records focusing exclusively on acute COVID-19 without reference to longer-term sequelae were excluded. Efforts were made to include studies with differing findings to minimise selective reporting bias. Control groups across included studies varied and comprised recovered SARS-CoV-2-infected individuals without persistent symptoms, uninfected controls, or combinations thereof. While the majority of studies analysed plasma or serum, a smaller subset examined cerebrospinal fluid (CSF), extracellular vesicles, or airway-derived samples.

**Table 1:** Search algorithms for proteomic landscapes of post-COVID condition.

Ovid MEDLINE(R). Dated 23/05/2026		
ID	Search terms	Records
1	exp COVID-19/or exp SARS-CoV-2/	320423
2	(“long COVID” or “post-COVID*” or PASC or “post-acute sequelae*” or PCC).ti,ab.	37853
3	1 and 2	13204
4	exp Proteomics/	86136
5	(proteomic* or proteome*).ti,ab.	168005
6	4 or 5	181193
7	3 and 6	103
8	limit 7 to (humans and yr=“2020 -Current” and journal article)	99
Link: https://ovidsp.ovid.com/ovidweb.cgi?T=JS&NEWS=N&PAGE=main&SHAREDSEARCHID=4Aj4oaqHuxImc1TKPHkaI66CsTbofHEESwvKPlC6ehRq0qmvjO8f1lKGkhjuSKghz		
Ovid Embase. Dated 23/05/2026		
ID	Search terms	Records
1	exp coronavirus disease 2019/or exp severe acute respiratory syndrome coronavirus 2/	548193
2	(“long COVID” or “post-COVID*” or PASC or “post-acute sequelae” or PCC).ti,ab.	52311
3	1 and 2	28839
4	exp proteomics/	176390
5	(proteomic* or proteome*).ti,ab.	220534
6	4 or 5	262959
7	3 and 6	272
8	limit 7 to (human and yr=“2020-Current” and article)	138
Link: https://ovidsp.ovid.com/ovidweb.cgi?T=JS&NEWS=N&PAGE=main&SHAREDSEARCHID=3jpy6K1P9dJNNeLh9wFSxvOErzDfTUHdtAb0S2IPVEMX0bWYBpoILCkE2Gm4YVoNG		

## Results and discussion

**Figure 1:**
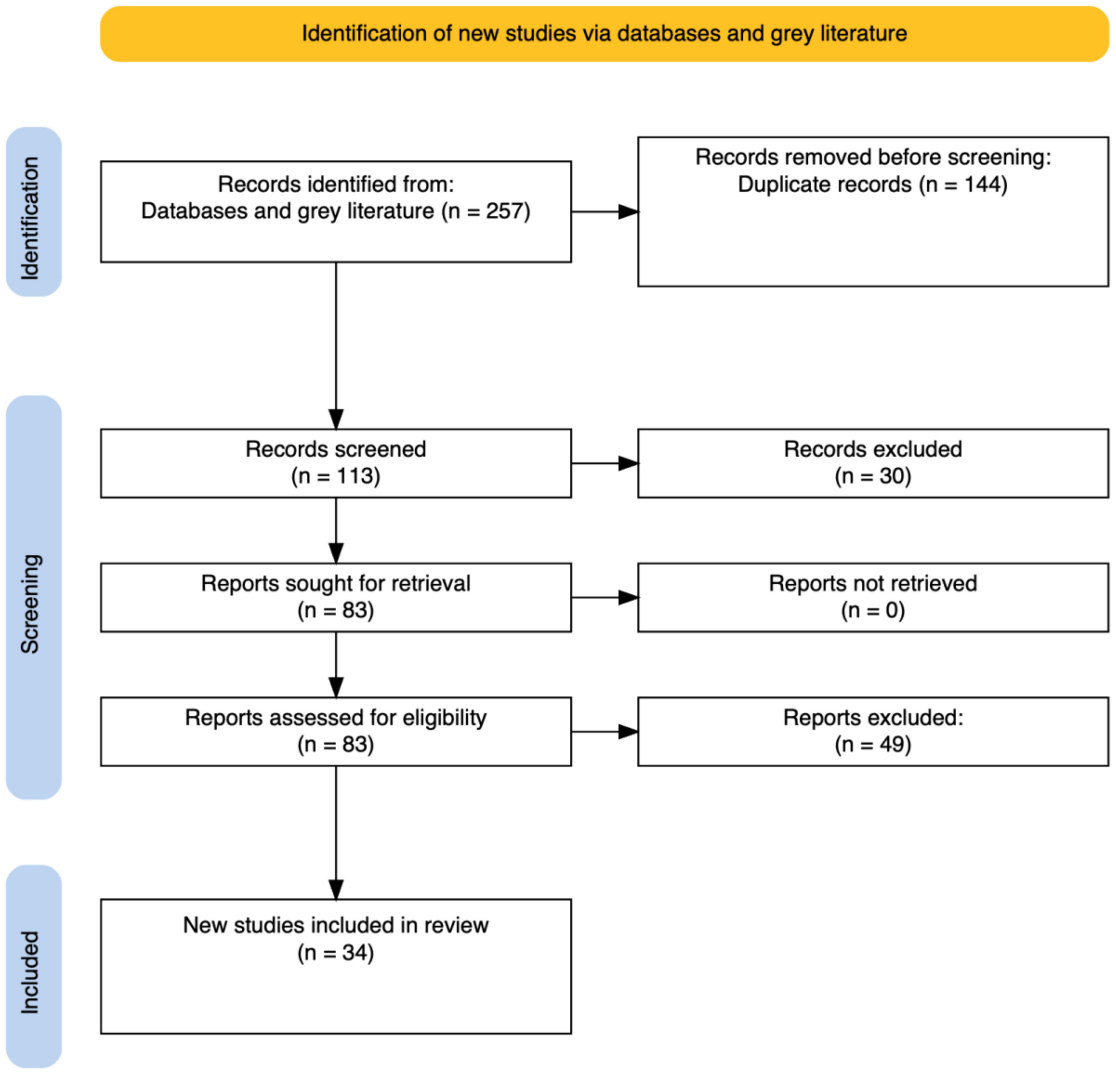
Study selection flowchart.

Out of 257 identified records (237 from two databases and 20 from grey literature) screened for eligibility, 34 were selected for inclusion (**Figure 1**). Where specific proteins were named, the most recurrently implicated domains include inflammation and cytokine signalling (IL-6, IL-1RN/IL-1RA, OSM, CCL4, CCL22, IFN-gamma, CX3CL1, CXCL9) (7–10), complement and coagulation cascades (C1 inhibitor, C3, C5, fibrinogen chains, alpha-2-antiplasmin, PF4, VWF, D-dimer, KNG1, CPB2) (9, 11–14), neutrophil-associated markers (ABCA13, CEACAM6, CRISP3, CTSG, BPI, elastase, citrullinated histone 3, NET markers) (15–17), oxidative stress and mitochondrial markers (PRDX6, VNN1, PON3, COX7A1) (11, 18), and vascular/angiogenic mediators (ANGPT1, VEGFA, ANGPTL2, TGFA) (7, 16).

## Thematic analyses

### Persistent immune dysregulation

Emerging evidence implicates persistent elevation of pro−inflammatory cytokines in PCC, notably interleukin-6 (IL−6), IL-20, C−type lectin domain family 10 member A (CLEC10A), monocyte chemoattractant protein 1 (MCP−1), tumour necrosis factor-alpha (TNF−α), and chemokine ligand 10 (CXCL10) (2, 4, 19–21). These mediators have been associated with symptom burden in several studies and may indicate chronic immune activation hypothesised to be contributing to fatigue and neurocognitive complaints (19, 20) through mechanisms including blood–brain barrier disruption, microglial activation, and sustained sickness-behaviour signalling. These proteomic signatures are not specific to PCC and more research is needed on mechanistic insights of these proteins and pathways in PCC. Additionally, emerging evidence suggests that extracellular vesicles may act as carriers of inflammatory and tissue-derived proteins in PCC, potentially enhancing detection of tissue-specific injury signals and mediating intercellular communication.

### Endothelial dysfunction and disordered haemostasis

The micro−clot and fibrinolysis−failure hypotheses have been implicated in PCC and are reported in several research studies (2, 13, 14, 22–25), although assay standardisation and independent replication remain variable. Some studies report fibrinolysis-resistant amyloid micro-clots enriched in α2−antiplasmin, fibrinogen chains, and serum amyloid A in PCC, accompanied by reduced kallikrein and elevated platelet factor 4 and von Willebrand factor, consistent with impaired fibrinolysis and platelet hyperactivation associated with fatigue, cognitive impairment, and dyspnoea (13, 14). Proteomic analyses further indicate disruption of the complement–coagulation–metabolic axis, with increased CPB2, KNG1, C1Q, and GAPDH, and differential expression of small extracellular vesicle proteins linked to complement activation alongside altered C1 inhibitor, C3, and C5 levels, suggesting that extracellular vesicles may capture tissue-derived signals not detectable in bulk plasma proteomics and may contribute to intercellular propagation of inflammatory signals (11, 12). However, these findings derive from specialised assays and require independent validation. Endothelial activation, including P−selectin and Intercellular Adhesion Molecule−1 (ICAM-1), angiogenic signalling via vascular endothelial growth factor A, and markers of coagulation and fibrinolysis such as von Willebrand factor and D−dimer, are associated persistent symptoms and impaired pulmonary function in PCC (22–25), including impaired gas transfer and radiological sequelae. These pathways may contribute to exertional intolerance and organ−specific hypoperfusion, although causal relationships cannot be inferred from currently available observational data.

### Neurological and pulmonary injury

Neuroaxonal and astrocytic markers, including neurofilament light chain, Glial Fibrillary Acidic Protein (GFAP), Hydroxyacylglutathione hydrolase (HAGH), N−acylethanolamine acid amidase (NAAA), and Latexin (LXN), are associated with neurological symptoms and inflammatory mediators in PCC, suggesting possible neuroinflammation−mediated injury (26–28) (limited consensus). Persistent spike protein accumulation has been reported along the skull–meninges–brain axis, accompanied by elevated CSF biomarkers of neurodegeneration and dysregulated inflammatory and neurodegenerative proteomic pathways (29). Certain differentially expressed CSF proteins have been identified in neuro-PCC, including increased ST1A1, with a diagnostic panel comprising sphinganine, 7,8-dihydroneopterin, and ST1A1 demonstrating high diagnostic utility (30). A neutrophil-upregulated PCC subtype has been associated with psychiatric manifestations (15), while specific autoantibodies have been linked to neuropsychiatric sequelae (31). Pulmonary−fibrotic signalling (TGF−β) is associated with radiological sequelae and reduced gas transfer in PCC, indicating a reparative−to−fibrotic shift in some patients (32, 33).

### From single markers to multi−analyte panels

Across studies, no single protein currently achieves translational diagnostic sensitivity and specificity. Most individual proteins identified (e.g., IL−6, TNF−α, endothelial markers) are not specific to PCC and are observed across post−viral syndromes, cardiometabolic disease, and chronic inflammatory states. However, their persistence beyond the acute phase and their co−occurrence across multiple dysregulated pathways may provide discriminatory value in specific PCC clinical phenotypes. I therefore advocate development and validation of multi−analyte panels mapped to clinical phenotypes (neurological, pulmonary, thrombo−inflammatory), combined with standardised sampling timepoints and harmonised assays (**Table 2**). The protein patterned clustering and chronic dysregulation, rather than individual proteins, may provide a better discriminatory value in PCC when compared with other clinical phenotypes.

**Table 2:** Phenotype-anchored multi-analyte panel for post-COVID condition clinical research.

Clinical phenotype	Proposed protein panel*	Clinical anchors/notes#	References
Neurological phenotype (brain fog, cognitive, headache)	NfL, GFAP, NAAA, LXN, HAGH, IL-6, IL-20, CXCL10A, MCP-1, ABCA13, CEACAM6, CRISP3, CTSG, BPI, COX7A1, ANGPT1, VEGFA, CCR7, CD56, citrullinated histone 3, elastase, D-dimer, IL-1RA, ICAM-1, sphinganine, ST1A1; spike protein persistence	Add cognitive battery (e.g., Montreal Cognitive Assessment (MoCA), reaction-time tasks etc.)	(4, 9, 15, 16, 18, 29, 30, 41)
Exertional intolerance/dyspnea	VEGF-A, vWF, ICAM-1, P-selectin, D-dimer, G-CSF, TRAIL, NBL1, CCL23, proteases, NET markers, HNRNPK, Haptoglobin, CD8 alpha chain, Vitamin D-binding protein, IgA heavy constant alpha 1, CXCL9	Pair with Cardiopulmonary Exercise Testing (CPET) and Diffusing Capacity of the Lungs for Carbon Monoxide (DLCO), and chest CT scoring	(4, 10, 17, 42–44)
Thrombo-inflammatory phenotype	IL-6, TNF-α, MCP-1, TRAIL, G-CSF, NBL1, CCL23, vWF, D-dimer, P-selectin, alpha-2-antiplasmin, fibrinogen chains, serum amyloid A (SAA), Kallikrein, PF4, ANGPT1, VEGFA, CCR7, CD56, citrullinated histone 3, elastase	Include thromboelastography or viscoelastic testing	(4, 13, 14, 16)

*These panels are conceptual frameworks derived from convergent signals across studies, rather than statistically derived or externally validated biomarker sets.

#Clinical anchors refer to objective, standardised functional or physiological measures used to contextualise biomarker findings (e.g., cognitive testing, Cardiopulmonary Exercise Testing (CPET) and Diffusing Capacity of the Lungs for Carbon Monoxide (DLCO)).

### Confounding variables

Vaccination status, reinfection episodes, and pre−existing comorbidities represent major sources of biological confounding in PCC proteomic studies, as each can independently induce long−lasting alterations in inflammatory, endothelial, and coagulation pathways. Failure to account for these variables may result in misattribution of nonspecific post−infectious or vaccine−related proteomic patterns to PCC−specific mechanisms. While vaccination attenuates acute inflammatory responses (34), recent vaccination or reinfection can produce transient immune and endothelial proteomic shifts that may overlap with PCC-associated pathways, making careful stratification by exposure history essential. Failing to stratify cohorts by vaccination status can obscure whether observed proteomic perturbations, such as those involving complement and coagulation pathways, are attributable to PCC, vaccine-induced immune signatures, or a hybrid of both (34, 35). In addition, literature suggests that proteomic signatures can remain altered months after infection (34), and longitudinal analyses are necessary to distinguish between transient immune activation due to recent reinfection and the persistent molecular dysregulation characterizing PCC. Furthermore, comorbid conditions, including cardiovascular disease, diabetes, and chronic kidney disease, can induce their own systemic protein signatures and PCC risk (36), which may confound the identification of PCC-specific biomarkers.

### Limitations

The rapid review approach facilitated timely synthesis; however, it is inherently constrained by scope and susceptible to selection bias. The majority of available data derive from high income countries and cross-sectional designs, limiting causal inference and introducing potential geographic and socioeconomic bias. Furthermore, many biomarker studies did not adjust for pre-existing conditions, sampling windows, batch effects, and assay heterogeneity or lacked validation in independent cohorts, raising concerns regarding the robustness of biomarker associations with PCC (1, 2, 4, 24, 27). Additionally, the reliance on peripheral proteomic measurements may not adequately reflect tissue-specific pathology, particularly for neurocognitive manifestations, where central nervous system or CSF sampling remains limited. These limitations highlight the urgent need for harmonised definitions, diverse tissue samples, longitudinal validation with pre−infection baseline, independent cohorts for validation, and global collaboration, rather than fragmented efforts conducted in isolation.

### Recommendations for PCC research

- Standardise and validate case definitions and sampling procedures, along with identification of promising biomarkers, for PCC clinical research (**Table 2**, **3**).
- Adopt standardised sampling windows (3, 6, 12 months) and covariate adjustment frameworks, where appropriate.
- Validate phenotype-anchored multi-analyte panels across platforms (e.g., Olink, MS, ELISA, etc.).
- Prioritise longitudinal proteomics and extracellular vesicle profiling for tissue-of-origin signals; prospects and pitfalls (37, 38).
- Validate candidate multi-analyte panels in independent cohorts and across platforms (19, 23).
- Integrate coagulation and endothelial assays to test micro-clot and endothelial hypotheses paired with functional outcomes such as Cardiopulmonary Exercise Testing (CPET) and Diffusing Capacity of the Lungs for Carbon Monoxide (DLCO) (24, 39).
- Several studies, including our previously reported cohort (2, 4), report divergent inflammatory and neurology−related protein patterns in PCC after primary and breakthrough infections; however, these observations require replication in fully independent, geographically diverse populations. Translational proteomics of PCC must actively incorporate diversity and inclusion to ensure that our molecular biomarkers and therapeutic targets are representative of all impacted communities in order to prevent worsening already-existing health inequities (40).
- The majority of available data derive from high−income countries and cross−sectional designs, limiting causal inference and raising concerns about transportability of candidate biomarkers across ancestries, healthcare systems, viral variants, and vaccination contexts. There is a critical need for future cross-population validation studies to ensure the global applicability of these biomarkers.

**Table 3:** Harmonising checklist for post-COVID proteomic clinical research studies.

Item	Considerations
Case definition	Adopt WHO Delphi PCC definition; report symptom onset and duration
Sampling window	Specify time since infection and/or vaccination (e.g., 3, 6, 12, 24 months) and fasting status
Assay harmonization	Report platform, calibration, Lower Limit of Quantification (LLOQ), Upper Limit of Quantification (ULOQ), batch effects, and spike-in controls
Covariate adjustment	Adjust for potential confounders such as age, sex, body mass index, comorbidities, vaccination/infection history, etc., where appropriate
Validation	Use independent cohort and cross-platform replication (e.g., Olink/MS/ELISA)
Clinical linkage	Pre-specify phenotypes and clinical anchors such as Cardiopulmonary Exercise Testing (CPET) and Diffusing Capacity of the Lungs for Carbon Monoxide (DLCO)

### Conclusion

A coordinated, phenotype−driven biomarker strategy, that is grounded in multi−analyte panels and prospective independent validation, represents a promising approach toward objective diagnosis and targeted interventions against PCC across diverse populations, healthcare settings, and geographic regions.
